# Incidental Learning of Temporal Structures Conforming to a Metrical Framework

**DOI:** 10.3389/fpsyg.2012.00294

**Published:** 2012-08-23

**Authors:** Melissa Brandon, Josephine Terry, Catherine J. Stevens, Barbara Tillmann

**Affiliations:** ^1^Marcs Institute, University of Western SydneySydney, NSW, Australia; ^2^Department of Psychology, University of Wisconsin – MadisonMadison, WI, USA; ^3^Department of Psychology, Florida Atlantic UniversityBoca Raton, FL, USA; ^4^School of Social Sciences and Psychology, University of Western SydneySydney, NSW, Australia; ^5^Auditory Cognition and Psychoacoustics Team, Lyon Neuroscience Research Center, CNRS-UMR 5292, INSERM U1028, Université de LyonLyon, France

**Keywords:** temporal cognition, metrical organization, serial reaction time task, implicit learning, incidental learning, auditory modality

## Abstract

Implicit learning of sequential structures has been investigated mostly for visual, spatial, or motor learning, but rarely for temporal structure learning. The few experiments investigating temporal structure learning have concluded that temporal structures can be learned only when coupled with another structural dimension, such as musical pitch or spatial location. In these studies, the temporal structures were without metrical organization and were dependent upon participants’ response times (Response-to-Stimulus Intervals). In our study, two experiments investigated temporal structure learning based on Inter-Onset-Intervals in the presence of an uncorrelated second dimension (ordinal structure) with metrically organized temporal structures. Our task was an adaptation of the classical Serial Reaction Time paradigm, using an implicit task in the auditory domain (syllable identification). Reaction times (RT) revealed that participants learned the temporal structures over the exposure blocks (decrease in RT) without a correlated ordinal dimension. The introduction of a test block with a novel temporal structure slowed RT and exemplified the typical implicit learning profile. Post-test results suggested that participants did not have explicit knowledge of the metrical temporal structures. These findings provide the first evidence of the learning of temporal structure with an uncorrelated ordinal structure, and set a foundation for further investigation of temporal cognition.

What do gymnastics, music, and language have in common? All three have sequential structure; events occur in a defined order (ordinal structure) with specific timing (temporal structure). For these activities, the order and the timing of the events create the distinct structure that distinguishes a gymnastics routine, a song, or a sentence from other instances of each of these activities. These examples illustrate that sequential structures are prevalent in everyday life, and that both the ordinal and temporal components are essential parts of the structure.

The acquisition of sequential structure has been studied extensively in the field of implicit learning. A classic experimental method used is the serial reaction time (SRT) task, developed to study visual-spatial sequence learning (Nissen and Bullemer, 1987). In this task, participants view a series of lights that are illuminated in a repeated sequence of locations (e.g., a 10-event sequence based on four possible locations), and respond by pressing the button corresponding to the location of each illumination. No explicit information is provided to participants about the prescribed location sequence. Evidence of learning the location sequence and the related motor responses are sought in the reaction time (RT) data: RT decreases with more exposure to the sequence, and increases when a novel sequence of locations is introduced. At the end of the experiment, participants are typically unable to identify or explicitly reproduce the learned sequence (Nissen and Bullemer, 1987). Over the years, this paradigm has been used to demonstrate that participants implicitly learn sequence information and they are not simply displaying perceptual-motor learning (see Tillmann and Poulin-Charronnat, 2010, for a review). The main focus in sequential structure learning research has been the learning of ordinal components of the sequential structure (e.g., Nissen and Bullemer, 1987; Reed and Johnson, 1994; Mayr, 1996; Saffran et al., 1996; Destrebecqz and Cleeremans, 2001; Olson et al., 2006), but there has been little research on the learning of the temporal components of sequential structure. The few extant studies have investigated temporal structure learning either using correlated ordinal structures or examining the interdependence of temporal and ordinal structures (Buchner and Steffens, 2001; Shin and Ivry, 2002; Ullén and Bengtsson, 2003; Karabanov and Ullén, 2008; O’Reilly et al., 2008).

Salidis (2001) adapted the SRT paradigm to assess adults’ implicit learning of rhythmic patterns. The participants’ task was to simply press a button every time they heard a beep. Thus, the ordinal sequence (the beep) was held constant and only the temporal component of this sequential structure was manipulated. The participants, however, were not informed that the presentation of the beeps followed a prescribed temporal pattern. Salidis (2001) reported learning (a) with larger RT decreases across blocks with structured temporal sequences in comparison to random temporal sequences, and (b) in comparison to the random exposure, the structured exposure led to an increase in RT for a randomly patterned test block, and finally, another decline in RT when the rhythmic pattern returned to the structured exposure sequence. This pattern of results was particularly pronounced for the shortest interval. Participants did not demonstrate any explicit knowledge of the rhythmic sequence in a subsequent production test and questionnaire. In Salidis’s study, the rhythmic structure was based on the response-to-stimulus interval (RSI), that is the interval between the participant’s response and the next beep. Timing structures based on the RSI, rather than the inter-onset interval (IOI), introduce variability in the temporal pattern due to the variability in the participant’s time to respond to each beep.

Salidis (2001) found temporal learning for auditory patterns that were six events long, and had simple symmetrical structures (e.g., 121,323, where the numbers represent multiples of a base interval duration). Other research has used longer rhythmic patterns (between 7 and 12 elements) without symmetric grouping, and directly compared the learning of ordinal and temporal structures. Both Shin and Ivry (2002) and O’Reilly et al. (2008) used visual SRT tasks to examine independent versus joint learning of temporal and ordinal structures in visuospatial sequences. Buchner and Steffens (2001) used an auditory SRT task to examine the correlated and uncorrelated learning of pitch and temporal sequences. In all three studies, no evidence of temporal structure learning was found in the presence of a random or uncorrelated ordinal structure; temporal structure learning was seen only when the temporal and ordinal patterns were perfectly correlated or systematically related. These results were obtained whether the temporal intervals were based on RSIs (Buchner and Steffens, 2001; Shin and Ivry, 2002) or IOIs (Shin and Ivry, 2002; O’Reilly et al., 2008). In light of their respective findings, some of the authors suggested that temporal learning might be facilitated by metric structure within the temporal patterns, such as found in music (Salidis, 2001; Shin and Ivry, 2002; Karabanov and Ullén, 2008). Our study investigates the learning of metrical temporal structures with an uncorrelated ordinal structure: the temporal structure is implemented with a series of spoken syllables presented in random order.

The temporal structure underlying Western tonal music is referred to as meter and is hierarchical in nature. A meter has a main pulse, or regularly spaced beat, that defines the intermediate level of the metric hierarchy, allowing for even subdivisions below the pulse and higher grouping above the pulse (Povel and Essens, 1985; Patel et al., 2005). A pattern is classified as strongly metrical if events in the pattern mostly occur on the beat, as opposed to having a silence on the beat or an event occurring on a subdivision of the beat, that is after the main pulse. Temporal patterns can have different metrical structures based on the grouping of events at the intermediate level between the main beats. For example, duple meters (DM) versus triple meters (TM) have events grouped in multiples of two versus three events, respectively. In the present study, we tested whether temporal patterns with metrical structures can be learned even without concurrent structure in the ordinal dimension. Syllable onsets marked the start of the temporal intervals, but the actual syllable presented was randomized. In addition, we investigated the influence of different metrical structures on temporal learning. Previous literature has demonstrated greater precision in perception and production performance of rhythms with duple, compared to triple meter (Smith and Cuddy, 1989; Drake, 1993; Desain and Honing, 2003). Hence, we compared learning of exposure patterns with duple versus triple metrical structures.

To test implicit learning of temporal patterns we used a modified version of the SRT task (Tillmann et al., 2011). In this task, participants listened to a series of syllables and identified each syllable (PA, TA, or KA) with a corresponding button press. The syllables themselves were presented in a random order, but the timing of the presentation of the syllables occurred according to a prescribed metrical structure (based on IOIs; Figure 1). The syllable identification task served as a cover story, enabling participants to make key presses following the timing patterns without directing their attention to these timing patterns. While the timing of the key presses was regular, which of the three keys they were pressing was unknown due to the randomization of the syllable order. In Experiment 1, participants were randomly assigned to either the DM or TM conditions and both conditions had a novel, DM pattern in the test block to assess learning. In Experiment 2, both exposure and test patterns were DM patterns, and the experimental material was controlled to ensure that RT changes were due to changes in the overall rhythmic structure rather than local changes between two intervals. In Experiments 1 and 2, participants responded to the exposure sequence for five blocks, and we hypothesized that the presence of the metrical structure created by controlling the IOIs of the syllable presentations would enable learning of the temporal patterns without a correlated ordinal structure (i.e., the order of the identity of the syllables did not follow a repeating structure). We expected to see evidence of learning with a decline in RT across the first five blocks, then an increase in RT in the test block (Block 6) when the novel pattern was introduced, and finally a return to the faster RT in the last block (Block 7), due to the return of the previously presented metrical pattern. Before the experimental phase, participants were not told about the timing aspect of the study. After the experimental phase, we assessed whether the participants had explicit knowledge of the temporal patterns from the experiment with a questionnaire and an explicit memory test (adapted from Destrebecqz and Cleeremans, 2001).

**Figure 1:**
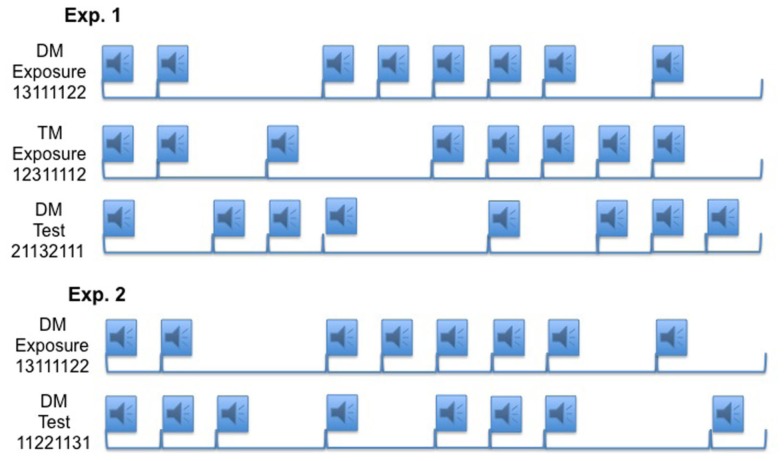
**A representation of the stimulus design, where the audio symbol indicates a syllable presentation (218 ms) and the brackets represent the assigned time intervals (interval times: 1 = 700 ms, 2 = 1400 ms, 3 = 2100 ms)**. For every syllable presentation one of three syllables (PA, TA, or KA) was presented according to a pseudo-randomized order. The exposure-timing pattern is presented for Blocks 1–5 and 7. The test pattern is presented in Block 6. There are 12 repetitions of the rhythm per block.

## Experiment 1

### Method

#### Participants

Forty-eight undergraduates or staff from the University of Western Sydney participated in the study for class credit or as a volunteer (38 females and 10 males, mean age = 21.5 years, age range = 18–52 years). Participants were randomly assigned to either the DM condition (*n* = 24) or the TM condition (*n* = 24). Participants in both conditions reported comparable musical training experience. The mean years of musical training for the DM condition was 3.42 years (±4.5), while the TM condition mean was 3.50 years (±4.6) with medians of 0 for the DM condition and 1 for the TM condition. The years of musical training of participants between conditions were not statistically different (*p* = 0.95). Participants were all right-handed and native English speakers, of which five were bilingual speakers. The University of Western Sydney Human Research Ethics Committee approved the experiment (HREC 07/006), and all participants read and signed an informed consent form.

#### Materials

##### SRT task

The syllables PA, TA, and KA were created using Mbrola, a text to sound speech synthesizer. The male voice “us3” was used at a pitch of 120 Hz. All syllables were 218 ms in length and were normalized for intensity. Pseudo-random syllable orders were created for the practice block, the five exposure blocks, the test block, and the return-to-exposure block with the following constraints linked to motor responses: (1) the three syllables occurred equally often, (2) no syllable was repeated in a row, and (3) the number of repetitions of a syllable on second and third orders (e.g., TA X TA = second order repetition, PA X X PA = third order repetition) was roughly balanced for each of the syllables. To control for syllable order effects that might create different levels of difficulty across blocks, we used a second order of syllables that was created by reversing the order from the initial syllable order, such that the final syllable of the final block became the first syllable of Block 1, and so forth, for all the syllables. These syllable orders were counterbalanced across participants.

The timing of the syllable presentations followed pre-assigned temporal patterns (based on IOI), thus independent of when the participant responded. To ensure the timing accuracy of stimuli presentation within each block, the syllables were concatenated off-line (using MATLAB) according to their prescribed metrical timing patterns and stored as auditory aiff files, creating one sound file per block with 12 repetitions of the temporal sequence in each block. PsyScope was used to present the auditory files and collect participants’ responses (Cohen et al., 1993).

The temporal patterns were selected from the metrically structured patterns of Povel and Essens (1985). In the following notation, two and three refer to intervals of duple and triple multiples of the basic temporal unit 1, which was 700 ms in this experiment. The exposure interval patterns were 13111122 (DM) and 12311112 (TM), and the test interval pattern was 21132111 (DM). All patterns consisted of five instances of the 1-interval, two instances of the 2-interval, and one instance of the 3-interval (Figure 1). Unlike the original Povel and Essens (1985) patterns, we removed the final long interval (4) from the temporal patterns. The long interval was omitted to prevent obvious segmentation of the pattern (with a starting point after the long interval). But without the consistent long interval at the end of the pattern, participants could potentially perceive the starting point of the sequence at different points or with groupings different from the original Povel and Essens (1985) patterns.

To assess which metrical structure participants perceived for each pattern, 10 musically trained participants performed a tapping task with the stimuli from Experiments 1 and 2 (five females and five males; mean age = 29.9 years, age range = 23–50 years; mean years of musical training = 13.8 years, SD = 9.5). Stimuli were six cycles (duration ∼50 s each) of each of the basic temporal patterns used in the SRT task: DM exposure (Experiment 1 and 2), TM exposure (Experiment 1), DM test (Experiment 1), and DM test (Experiment 2; refer to the Method section of Experiment 2 for further details). The sequences were constructed using a piano tone with a pitch of C4 (261.6 Hz). All patterns were presented twice to each participant in a randomized order. Participants listened to each sequence over headphones and when ready, tapped what they believed to be the best-fitting meter. They continued tapping until the end of the sequence. Taps were made on a table and were recorded by a microphone (AKG condenser C391B) generating a single stereo file with tapping on one channel and the presented sequence on the other channel. The temporal onsets of the taps relative to the piano tones were identified and used to analyze participants’ metrical interpretation of each sequence. The results confirmed that the DM exposure and DM test patterns for Experiment 1 were primarily perceived with a DM (84 and 74% of trials, respectively), while the TM exposure pattern was primarily perceived with a TM (70% of trials).

##### Post-test

The stimuli used for the explicit memory task were the three metrical patterns used in the experimental task (the two exposure patterns and the test pattern), as well as two novel patterns that served as foils for the exposure patterns: 12121113 and 11122311. The foil patterns each had at least two bigrams (i.e., two intervals directly following each other) in common with the exposure patterns.

The patterns in the explicit memory task had a base unit interval of 700 ms, just as the patterns in the experimental task. Instead of syllables, the metrical patterns were constructed from a piano tone at the pitch of C4. Piano tones were used to direct the participants’ attention to the timing of the events and prevent them from basing their decisions on familiarity with the syllables, their chaining, or their timbre/spectral content. The patterns were created using Max MSP, which controlled the pattern timing and was interfaced with a MIDI system to generate the 218 ms grand piano tone. The program produced audio files of each metrical pattern. Because it was possible that the participants could have grouped the patterns from different starting points, we presented each pattern with three different starting points. In total, there were 15 post-test trials, three trials for each pattern in the post-test.

#### Procedure

##### SRT task

Participants listened to a randomized sequence of syllables (PA, TA, or KA) over headphones and were instructed to identify each syllable by pressing the corresponding key on the keyboard as quickly and accurately as possible (i.e., three-alternative forced choice task); no feedback was given. Participants were told that if they missed a syllable or made an incorrect response, they should not correct themselves. Number keys 1, 2, and 3 on the number keypad of a computer keyboard (labeled above with the attributed syllables) were used to collect participants’ responses and RT. The key-to-syllable mappings were counterbalanced across participants. Participants were instructed to keep their index, middle, and ring fingers of their right-hand on the response keys at all times. The participants began with a practice block of 24 syllables, which followed the temporal pattern of the exposure phase. Participants then completed seven blocks with 96 syllables in each block, separated by short breaks (on average 1 min). Blocks 1–5 were the exposure blocks. Block 6 was the test block introducing the novel pattern, followed by a return to the exposure pattern in Block 7.

##### Post-test

After the SRT task, participants completed a written questionnaire about the task (adapted from Salidis, 2001). Participants described what they thought the task was about, if they had noticed any regularities in the task material, and what strategies they had used. They were also asked to decide which of the final two blocks of trials was more like the rest of the task and rate their confidence in this decision using a subjective scale from 1 = not confident, over 4 = somewhat confident, to 7 = very confident. After collecting the questionnaire, the experimenter informed participants that there was regularity in the timing of the syllables. The participants then completed a computerized recognition post-test to assess their explicit knowledge of the metrical timing patterns encountered during the SRT task. Participants were told to listen to the piano sequence and then, using a 6-point scale, judge the familiarity of the piano sequence’s timing compared to that of the syllable’s timing they had been hearing during the first task of the experiment. The scale (taken from the post-test of Destrebecqz and Cleeremans, 2001) was as follows: 1 = Certain the sequence was Not in the study, 2 = Fairly certain the sequence was Not in the study, 3 = Believe the sequence was Not in the study, 4 = Believe the sequence Was in the study, 5 = Fairly certain the sequence Was in the study, 6 = Certain the sequence Was in study.

Given that some of the post-test patterns started or ended with silent intervals an indicator of the total length of the temporal sequence was needed. The participants were informed that at the start of the temporal sequence the entire computer screen would change to a bright green color and stay green until the end of the sequence. After the piano tone sequence was presented and the screen returned to black, the rating scale appeared on the screen for participants to rate the familiarity of the temporal sequence. The scale stayed on the screen until participants responded by pressing the number key corresponding to their rating, then participants advanced to the next trial by pressing the space bar. At the conclusion of the study, participants completed a demographics and music experience questionnaire and were debriefed. The experiment took 45 min.

### Results

#### SRT task

For each syllable, the response time window was defined to be a response occurring between 100 and 800 ms from syllable onset. Mean response times for correct responses were analyzed per block for each participant, collapsed across syllables. For the TM group, one participant was excluded from the analyses for low accuracy (22.77%). For the remaining participants, the TM exposure group’s overall accuracy was 81.19 ± 10.24% and the DM exposure group’s accuracy was 73.43 ± 14.45%.

**Figure 2:**
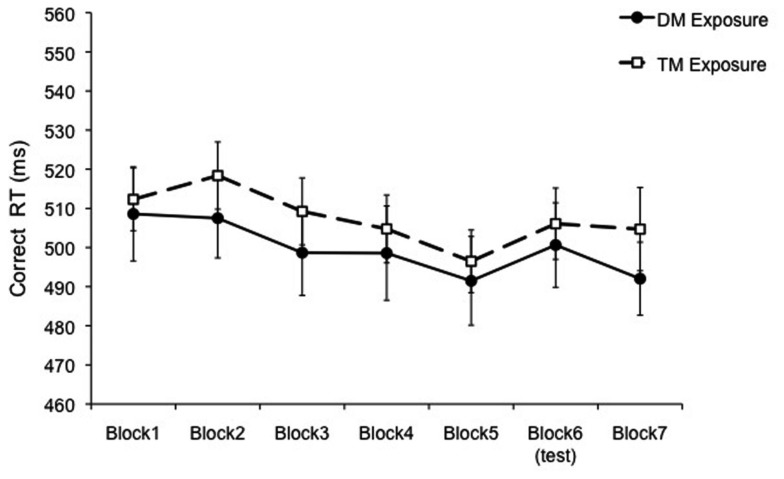
**Mean correct RT for Experiment 1 presented as a function of block separated by exposure group (Duple Meter or Triple Meter)**. The test block was Duple Meter. Error bars represent the standard error of the mean.

An ANOVA with Block (seven levels) as a within-subject factor and Exposure (DM or TM exposure patterns) as a between-subjects factor was run on the correct RT data (Figure 2). The main effect of Block was significant, *F*(6, 270) = 4.26, *p* = 0.001, MSE = 473.94 [Greenhouse–Geisser correction, *F*(4.71, 212.09), *p* = 0.001], but there was no main effect of Exposure and no interaction, *p*s > 0.55. RT decreased significantly from Block 1 to Block 5, *F*(1, 45) = 8.74, *p* = 0.005, and increased from Block 5 to Block 6, with the introduction of the test block, *F*(1, 45) = 4.28, *p* = 0.04. The re-introduction of the exposure sequence decreased RT, but not significantly, *p* = 0.19. This lack of recovery in the final block has been observed in previous SRT studies, leading some researchers to implement multiple exposure pattern blocks after the test block to enable recovery of RT speed (Buchner and Steffens, 2001; Destrebecqz and Cleeremans, 2001; Shin and Ivry, 2002; O’Reilly et al., 2008). Due to our long temporal patterns and the overall length of our experiment, we did not add an additional block of the exposure pattern at the end.

Another measure that has been used in previous SRT research to assess learning compares the RT of the test bock to the average RT of the exposure blocks preceding and following the test block (i.e., learning score; Salidis, 2001). In our experiment, this comparison demonstrated learning, with a significantly shorter RT for the mean of Blocks 5 and 7 compared to Block 6, *F*(1, 45) = 4.29, *p* = 0.04.

**Figure 3:**
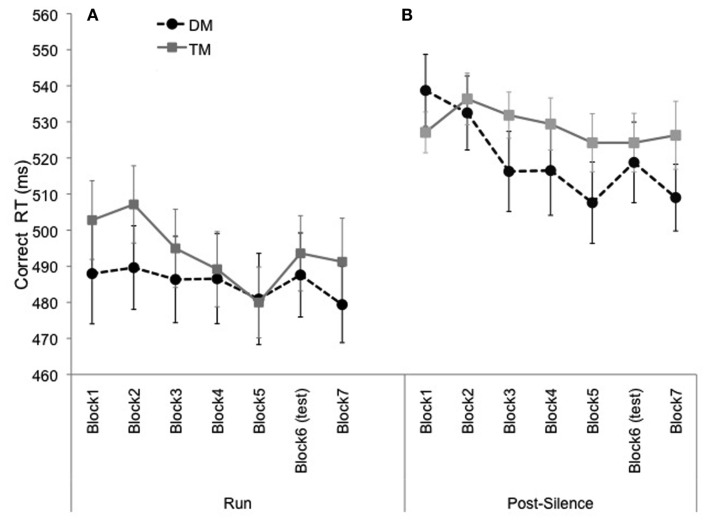
**Mean correct RT for Experiment 1 presented as a function of block separated by event-type (run vs. post-silence)**. **(A)** Are the run events and **(B)** are the post-silence events. Error bars represent the standard error of the mean.

In Salidis (2001), the increase in RT for the test block was particularly pronounced for the short intervals. Therefore, we performed this same analysis for our data. We separated responses that followed another event directly (e.g., XX), referred to as “run” events, from responses that followed a silent event (e.g., X.X or X.X), referred to as “post-silence” events (Figure 3). An ANOVA with Type (run/post-silence) and Block (seven levels) as within-subject factors and Exposure (DM/TM) as a between-subjects factor revealed a main effect of Type, *F*(1, 45) = 72.18, *p* < 0.001, MSE = 2697.19. RT was overall faster for the run events than for the post-silence events. The main effect of Block was significant, *F*(6, 270) = 4.28, *p* < 0.001, MSE = 919.82 [Greenhouse–Geisser correction, *F*(4.74, 213.44), *p* = 0.001], in addition, there was a significant three-way interaction between Type, Block, and Exposure, *F*(6, 270) = 2.96, *p* = 0.008, MSE = 455.28. Follow-up tests were conducted to examine these main effects and interactions for learning. With DM exposure, RT decreased from Block 1 to 5 for the post-silence events, *F*(1, 45) = 15.22, *p* < 0.001, while for the run events (i.e., short intervals), RT was fast from the start of the experiment and thus did not significantly decrease through Block 5 (*p* = 0.46). In contrast, after TM exposure, RT to run events in Block 1 was slower than for the DM exposure group and decreased significantly through Block 5, *F*(1, 45) = 5.63, *p* = 0.02, reaching the speed of the DM group. However, for the post-silence events, the TM sequences did not enable participants to speed up over Block 1 to Block 5, *p* = 0.73.

Regarding the introduction of the test sequence in Block 6 there are some indications of learning, but separating run and post-silence events around the test block also led to decreased statistical power in each condition. For both DM and TM groups indications of learning can be observed for the run events with RT slowing down in Block 6, even if this RT change failed to reach significance, *F*(1, 45) = 2.89, *p* = 0.096. For the post-silence events, only the DM exposure group demonstrated slower RT for Block 6 in comparison to the surrounding exposure blocks, even if this learning score fell short of significance, *F*(1, 45) = 3.17, *p* = 0.08. For the post-silence events, the RT of the TM exposure group was rather slow overall and remained stable over all the blocks.

#### Post-test

Participants’ responses to the written questionnaire suggest very few participants had any explicit knowledge that the purpose of the task was about learning the timing of the syllable presentations. In response to the question “what regularities did you notice in the materials,” the most frequent response was that there was a pattern in the order of the syllables, with 25 of the 47 participants responding incorrectly in this manner (12 of the 24 DM condition participants and 13 of the 23 TM condition participants). Only 7 of the 47 participants mentioned anything related to the timing of the presentation of the syllables (e.g., “noticed some rhythm in the sequences”). Breaking this down by condition, 5 of the 24 participants in the DM condition and 2 of the 23 participants in the TM condition mentioned any regularity in the timing of the syllables.

When participants had to rate which of the final two blocks of trials was more like the rest of the task, only 29 of the 47 participants (61.70%) correctly identified the final block; this was not statistically different from chance (*p* = 0.11). Likewise, when broken down by condition, only 16 of the 24 DM (66.67%) and 13 of the 23 TM (56.52%) condition participants correctly identified the final block, and neither group’s performance was different from chance (DM: *X*^2^ (1, 24) = 2.667, *p* = 0.102; TM: *X*^2^(1, 23) = 0.391, *p* = 0.532). Finally, the participants were not overly confident about their choice, with a mean confidence rating of 3.13 (SD = 1.61; split by exposure group: DM = 3.42 (SD = 1.56) and TM = 2.83 (SD = 1.64), and these mean confidence ratings did not differ across groups [*t*(45) = 1.265, *p* = 0.212)].

Results from the recognition test provided further evidence that participants did not have explicit knowledge of the temporal information. All of the mean ratings fell between 3 and 4 on the scale, which indicates that the participants were relatively uncertain about their familiarity with the patterns. In the post-test, the participants rated three different novel patterns (the two foil patterns and the exposure pattern of the other group). To investigate potential differences between the novel patterns, a 3 × 2 ANOVA was conducted with the three novel patterns as the within-subject factor and the assigned exposure as the between-subjects factor. No significant main effects or interactions were observed, *p*s > 0.175. With no difference between the familiarity ratings for the novel patterns, the mean of these three patterns was used as the Novel category in the main analysis of the post-test ratings. The familiarity ratings were thus grouped into three categories: Exposure, Test, and Novel (Table 1), and analyzed as the within-subject factor in a 3 × 2 ANOVA with Exposure (DM vs. TM) as the between-subjects factor. No significant main effects or interactions were observed, *p*s > 0.067. The participants did not rate the patterns to which they had been exposed as more familiar than the patterns that they had not encountered.

**Table 1:** **Mean ratings and SD for each category of post-test ratings by exposure group**.

Category	Duple meter exposure (Experiment 1)		Triple meter exposure (Experiment 1)		Duple meter exposure (Experiment 2)	
	*M*	SD	*M*	SD	*M*	SD
Exposure	3.74	0.75	3.91	0.72	4.06	0.75
Test	3.54	0.96	3.58	0.79	3.90	0.93
Novel	3.44	0.62	3.55	0.52	3.74	0.63

### Discussion

The aim of Experiment 1 was to investigate the incidental learning of temporal patterns with metrical structure when the associated ordinal information was random. Our results revealed that participants learned the metrically organized temporal patterns, regardless of the metrical grouping. The overall RT data reflected the typical learning profile seen in an SRT task: a significant decrease in RT across the exposure phase, and a significant increase in RT when a novel temporal pattern was introduced. The increased RT in response to the novel temporal pattern in Block 6, when all other aspects of the task remained the same, was evidence of learning the temporal patterns beyond task or motor-related learning. Furthermore, the post-test data indicated that participants’ knowledge of the temporal patterns was not explicit.

Interestingly, the two metrical grouping conditions did not show different learning patterns unless they were broken down by run and post-silence events. For the post-silence events, which should be the most difficult events to predict because of the longer temporal interval (e.g., Eisler et al., 2008), participants in the DM exposure group successfully demonstrated learning of the temporal pattern: RT decreased significantly over exposure blocks and increased for the test block (just falling short of significance). However, this learning of post-silence event timing was not observed for the TM exposure group. These findings suggest that the DM context enabled participants to form a metrical grid underlying the temporal events, aiding anticipation of the longer event timing (post-silence events). By contrast, the TM context did not support this same learning advantage for the post-silence events. Nevertheless, the exposure to the TM pattern still allowed participants to increase speed of RT for run events, and to reach the speed of the DM exposure group by block 5. These patterns of results thus suggest that temporal pattern learning did occur in both the DM and TM contexts, but that the different meters affected the temporal learning in different ways as seen by the differences in the run/post-silence analysis.

For both DM and TM conditions in Experiment 1, the test pattern contained a new interval transition that had not occurred in the exposure pattern (Figure 1). In the DM exposure pattern, the 3-interval was followed by a 1-interval, leading to the transition 31, but the test pattern introduced the new transition 32. Consequently, participants’ expectations may have been violated by this new local interval transition, resulting in a longer response time following the 2-interval in the test. Similarly, the test pattern introduced two new transitions that did not occur in the TM exposure pattern, notably 13 and 32. Even though it seems unlikely that these new interval transitions might explain the observed learning effects because they represent only one or two out of eight possible transitions, Experiment 2 used a test pattern that did not introduce any new transitions. Experiment 2, thus, controlled for the frequency of occurrence of the intervals (as in Experiment 1), as well as the transitions across intervals on a bigram level (i.e., two intervals directly following each other). This subtle manipulation allowed scrutiny of whether participants learned temporal regularities beyond the local level of two intervals, requiring learning of at least three intervals (i.e., the chaining of four events).

## Experiment 2

### Method

#### Participants

Twenty-five undergraduates from the University of Western Sydney participated in the study for class credit (24 females and 1 male, mean age = 21 years, age range = 18–36 years). Participants were all right-handed and native English speakers, of which two were bilingual speakers. The mean years of musical training was 0.26 years (±0.52), with a median of 0. The University of Western Sydney Human Research Ethics Committee approved the experiment (HREC 07/006), and all participants read and signed an informed consent.

#### Materials

##### SRT task

The temporal patterns were constructed using the same procedure and protocol as outlined in Experiment 1 (and adapted from patterns reported in Povel and Essens, 1985). The exposure pattern was the same DM pattern used in Experiment 1, and the test pattern was an IOI sequence of 11221131 (Figure 1). These two patterns kept constant the interval transitions between exposure and test patterns, such that the changes in RT in the test block could not be attributed to changes in local interval co-occurrences. The metrical interpretation of the exposure pattern was confirmed as DM in the tapping task reported in the Method section of Experiment 1. The primarily perceived meter for the test pattern was duple (65% of trials).

##### Post-test

The stimuli used for the explicit memory task were the two DM patterns used in the experimental task (exposure and test), as well as the novel patterns that had been used in Experiment 1 as foils. The trial construction and stimulus parameters were as described in Experiment 1.

#### Procedure

The procedure was identical to that described for Experiment 1.

### Results

#### SRT task

Correct responses and correct response times were analyzed, as in Experiment 1. One participant was excluded from the analyses for low accuracy (30.06%). For the remaining participants, overall accuracy was 70.24 ± 11.41%.

**Figure 4:**
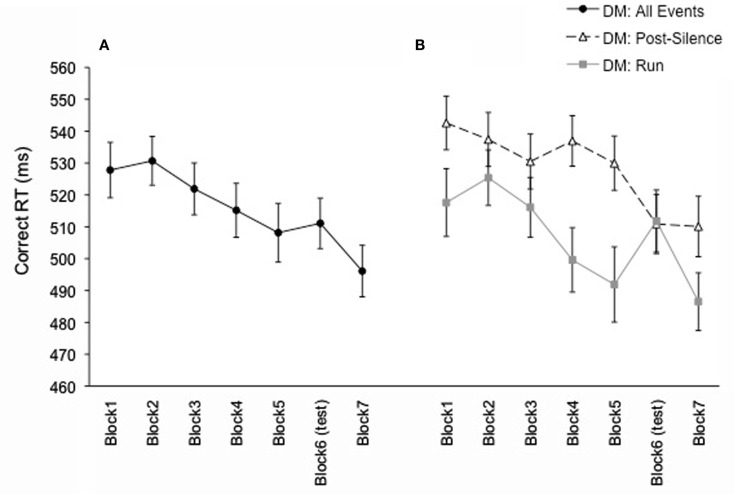
**Mean correct RT for Experiment 2 (exposure and test block were Duple Meter), presented as a function of block in (A) and as a function of block separated by event-type (run vs. post-silence) in (B)**. Error bars represent the standard error of the mean.

Correct RT data were analyzed in a one-way ANOVA with Block (seven levels) as the within-subject factor (Figure 4A). This analysis revealed a significant main effect of Block, *F*(6, 138) = 5.33, *p* = 0.001, MSE = 654.07 [Greenhouse–Geisser correction, *F*(4.01, 92.28), *p* = 0.001]. RT decreased significantly from Block 1 to Block 5, *F*(1, 23) = 4.67, *p* = 0.04. RT did not increase with the introduction of the test block (*p* = 0.60), but did decrease significantly with the return of the exposure sequence in Block 7, *F*(1, 23) = 6.35, *p* = 0.02. The learning score (comparing the RT of the test block to the average RT of exposure blocks preceding and following the test block) fell just short of significance, *F*(1, 23) = 3.92, *p* = 0.06.

Again, the analysis examining run and post-silence event learning was performed (as in Salidis, 2001 and Experiment 1). An ANOVA with Type (run/post-silence) and Block (seven levels) as within-subject factors revealed a main effect of Type, *F*(1, 23) = 11.25, *p* = 0.003, MSE = 3402.96, with overall faster RT for run events than post-silence events. The main effect of Block was significant, *F*(6, 138) = 5.35, *p* = 0.001, MSE = 1248.38 [Greenhouse–Geisser correction, *F*(4.11, 94.42), *p* = 0.001], as was the interaction between Type and Block, *F*(6, 138) = 3.92, *p* = 0.001, MSE = 604.42. From Block 1 to Block 5, RT decreased for both Types, but more strongly (i.e., steeper slope) for run events than for post-silence events (see Figure 4B). For run events, RT increased between Block 5 and Block 6 (the test block), *F*(1, 23) = 4.63, *p* = 0.04, and decreased with the return to the exposure sequence in Block 7, *F*(1, 23) = 13.09, *p* = 0.001. The learning score was also significant for the run events, *F*(1, 23) = 11.29, *p* = 0.003. For post-silence events, however, RT continued to decrease through Block 6, *F*(1, 23) = 10.86, *p* = 0.003, and then remained stable for Block 7, *p* = 0.93.

#### Post-test

Consistent with Experiment 1, the written questionnaire responses suggest few participants had gained explicit knowledge about the purpose of the task. For the open response question about regularities in the materials, the most frequent response was that there was a pattern in the order of the syllables with 12 of the 24 participants responding incorrectly in this manner. Only three participants mentioned anything related to the timing of the syllables.

When participants rated which of the final two blocks was more like the other blocks, 13 of the 24 correctly identified the final block, but this was not statistically different from chance (*p* = 0.221). Participants were only “somewhat confident” (see scale definition in the method section) about their choice with a mean confidence rating of 4.13 (SD = 1.15).

From the recognition test, means were calculated for the familiarity ratings of the three variants of each pattern, as in Experiment 1. All mean ratings fell between three and four on the scale, within the range of uncertainty. A *t*-test compared the two foil patterns [*t*(23) = 0.726, *p* = 0.475], assuring that they were not statistically different from one another before they were averaged to form the Novel category for the overall post-test analysis. The mean ratings of familiarity were then analyzed in three categories: Exposure, Test, and Novel (the average of the ratings for the two foil patterns; Table 1), as a within-subject ANOVA, which did not reveal a significant effect (*p* > 0.245). These findings from the post-test were consistent with the post-test results for Experiment 1, and suggest that participants did not have explicit knowledge of the learned timing pattern.

### Discussion

Experiment 2 investigated implicit learning of a temporal pattern with a duple metrical framework by using a test pattern that also had a DM and no changes in the local interval patterns across the exposure and test sequences. The changes in the interval patterns were at a higher level, requiring learning of patterns at least three intervals long. The overall results showed a decrease of RT over exposure blocks (Blocks 1–5), replicating the learning over the exposure phase observed in Experiment 1. In contrast to Experiment 1, where RT increased with the introduction of the test pattern and decreased with the return of the exposure pattern, the introduction of the test pattern in Experiment 2 elicited a different response. The test block RT remained stable relative to the exposure blocks, rather than slowing down as in Experiment 1. It is the return to the exposure pattern after the test block that did provide a benefit and elicited a significantly faster RT in Block 7 (Figure 4A). Participants’ learning in Experiment 2 was more clearly observed when the data were broken down by run/post-silence events.

For Experiment 2 (as in Salidis, 2001 and Experiment 1), the RT data were separated as a function of the preceding interval (a short interval referred to as “run” and longer intervals referred to as “post-silence”), providing further insights into the learning of temporal patterns with a metrical framework. Overall, RT was faster for run events than for post-silence events, indicating less difficulty in processing shorter intervals than longer intervals (as in Experiment 1). Most importantly, the data from the run events revealed the classical SRT learning pattern: RT increased in the test block and decreased with the return to the exposure pattern. This finding suggests that temporal pattern learning occurred at least for the short intervals. In addition, the controlled construction of exposure and test patterns allows us to conclude that learning goes beyond the transitions of two intervals (i.e., interval bigrams), as these were kept constant between exposure and test. The test pattern only introduced new interval transitions at the trigram or quadrigram level. The structure of the temporal patterns and the increased RT for the test pattern indicate that participants predicted the next events on the basis of learning at least three preceding intervals.

For post-silence events, however, the RT in the test block continued to decrease, and then leveled off in the final block with the return of the exposure pattern. While this is an atypical behavior profile for an implicit learning task, we suggest that this pattern reflects the influence of both the consistency of the metrical framework and the maintenance of bigram interval transitions across the exposure and test patterns. The DM patterns used here likely activated a metrical grid that enabled participants to learn the longer, and more difficult intervals. As shown by previous research on metrical pattern processing (e.g., Large and Kolen, 1994), a metrical grid helps listeners to anticipate the next beat in an auditory pattern, and in the current experimental paradigm the potential temporal position of the next syllable. This temporal prediction based on (or assisted by) the metrical grid becomes particularly relevant for the prediction of longer temporal intervals, which are more difficult to process (e.g., Eisler et al., 2008). In Experiment 2, the metrical framework continued to benefit temporal prediction in the test block when bigram transitions were maintained. In other words, the participants had already experienced all possible local interval transitions during the exposure phase so they just had to predict when in the test pattern they would occur, aided by the continued DM context.

As for the leveling off observed in Block 7 for the post-silence events, rather than an RT decrease, the plateau can most likely be attributed to the impact of two changed temporal patterns in a row. In some SRT studies (e.g., Shin and Ivry, 2002; O’Reilly et al., 2008), participants completed multiple test blocks that were separated by three to five blocks of the exposure pattern to enable recovery of the RT between the test blocks. For our study, we did not add a second exposure block after the test block, because of concerns for participants’ attentional fatigue from this demanding speeded syllable discrimination task, when they still needed to complete the post-test tasks. Future experiments in our line of study might thus integrate multiple exposure blocks in order to allow for recovery of the RT after the disturbance created by the test block.

Overall, our findings support the idea that implicit learning of the temporal patterns occurred in the face of unchanging metrical structure and uncorrelated ordinal patterns of the syllables. Finally, the post-test results, in agreement with Experiment 1, indicated that the temporal learning occurred implicitly.

## General Discussion

The goal of our study was to investigate the incidental learning of metrical temporal patterns in the context of sequential structures with uncorrelated ordinal patterns. In previous research, temporal pattern learning did not occur in the presence of uncorrelated ordinal structure, but occurred only when temporal and ordinal patterns were systematically related (e.g., Buchner and Steffens, 2001; Shin and Ivry, 2002). Temporal learning was evident in results from Salidis (2001) without concurrent ordinal structures (a repeated tone). However, the Salidis (2001) study used only six-event symmetric sequences. Our results provide evidence for incidental learning of more complex temporal patterns constructed from spoken syllables, in which the syllable order was not correlated with the temporal structures. A major difference from previous studies was the temporal organization of the stimuli. The use of temporal intervals based on IOIs (instead of RSIs) ensured that the temporal intervals remained constant over pattern repetition and were not subject to the variability of participants’ response times. This also allowed for the establishment of a metrical framework, which appears to be the organization necessary for the incidental learning of temporal structure in the presence of an uncorrelated ordinal structure.

Additionally, the results demonstrate temporal learning for metrically rhythmic patterns with either duple or triple meter. In Experiment 1, overall RT decreased significantly over exposure blocks and increased during the test block for both DM and TM exposures. When the data were separated into run and post-silence events, a difference between the DM and TM conditions was found. Learning of post-silence event timing was observed for the DM, but not the TM exposure group. This is consistent with previous literature demonstrating better perception and production performance of rhythms with duple, compared to TMs (Smith and Cuddy, 1989; Drake, 1993; Desain and Honing, 2003). In Experiment 2, while overall RT decreased significantly from Block 1 to 5, RT increased during the test block for run events only. Hence, like Salidis (2001), we observed the learning effect mostly for the run events. However, in the DM condition of Experiment 1 and Blocks 1–5 of Experiment 2 incidental learning was also evident for the post-silence events. These findings were most likely due to the activation of a metrical grid, which facilitates the anticipation of the long intervals that are more difficult to predict (Eisler et al., 2008). Our findings not only build on, but also extend those of Salidis (2001): our temporal patterns consisted of eight elements (instead of six), and they were characteristic of musical structure (e.g., Povel and Essens, 1985) instead of being two symmetric triplets (121,323). Furthermore, we showed temporal learning not for a single, repeated tone, but for patterns based on an uncorrelated ordinal dimension that was unstructured (the random presentation of syllables).

The stimuli construction also controlled for several features of the temporal structures across exposure and test sequences. Thus, in asking what has been learned, we can be confident that the increased RTs observed in the test blocks were not due to changes in interval lengths as the intervals used in exposure and test patterns were the same. Also, the frequencies of occurrence for all intervals in exposure and test blocks were the same. With regard to the chaining of intervals, local transitions of interval bigrams were kept constant between exposure and test blocks in Experiment 2. Taking the results of Experiments 1 and 2 together, we conclude that the temporal learning observed here goes beyond local temporal transitions to higher temporal regularities.

In Experiments 1 and 2, the syllable identification task drew participants’ attention to features other than time. The results from the questionnaire and recognition post-test in both experiments suggested that participants did not acquire explicit temporal pattern knowledge. Our findings are in accord with Salidis (2001) who tested implicit knowledge not only with post-SRT-task interviews, but also with an adaptation of a generation task (i.e., participants used numbers, each representing one of the three intervals, to indicate the order of the intervals in the repeating sequence). Future studies need to further develop the generation task for the temporal dimension in order to allow for more specific testing of the learned structures. Together, Salidis (2001) and our results support implicit cognition as a means for temporal learning.

Compared to other studies on implicit temporal learning (e.g., Shin and Ivry, 2002) the tempo of our rhythmic stimuli may be considered rather slow. The slow tempo was chosen to allow for reasonable accuracy in the syllable identification task. It is worth noting that even though the relatively slow tempo resulted in the total timing of 8.4 s per sequence, the data pattern clearly showed temporal learning across the exposure blocks. Our findings along with other recent studies demonstrating the implicit learning of long sequences, such as dance movement sequences of lengths up to 19 s (Opacic et al., 2009), support the idea that implicit learning is not limited by working memory constraints (e.g., Frensch and Miner, 1994).

The present study provides a foundation for investigating the advantage afforded by metrical organization for the incidental learning of complex temporal structures. While temporal learning with uncorrelated ordinal structures has not been demonstrated in previous SRT studies (Buchner and Steffens, 2001; Shin and Ivry, 2002), the metrical grid underlying the present stimuli is likely a key feature for temporal structure learning. Future studies need to compare metrical structures with non-metrical structures to further demonstrate the processing advantage of metrical structure in temporal learning. The present paradigm and results also call for further investigation of the capacity of implicit temporal cognition in the learning of sequential structure, notably (a) the use of more complex temporal patterns (longer than eight events), (b) the use of less familiar meters (such as 7/8 seen in non-western music), and (c) the introduction of regularities in the ordinal dimension, which are either correlated or uncorrelated with the regularities in the temporal dimension.

## Conflict of Interest Statement

The authors declare that the research was conducted in the absence of any commercial or financial relationships that could be construed as a potential conflict of interest.
